# Effects of mature cystic teratoma on reproductive health and malignant transformation: A retrospective analysis of 80 cases

**DOI:** 10.4274/jtgga.galenos.2018.2018.0003

**Published:** 2019-05-28

**Authors:** Sefa Kurt, Hüseyin Aytuğ Avşar, Ömer Erbil Doğan, Hasan Bahadır Saatli, Uğur Saygılı

**Affiliations:** 1 Department of Obstetrics and Gynecology, Dokuz Eylul University School of Medicine, İzmir, Turkey

**Keywords:** Mature cystic teratoma, malignant degeneration, infertility treatment

## Abstract

**Objective:**

To examine cases of mature cystic teratoma (MCT) that were diagnosed and treated in our clinic regarding their association with fertility, and to detect the rate of malignant degeneration and the types of malignancies.

**Material and Methods:**

Patients who underwent surgery due to adnexal mass between April 2012 and August 2017 and were diagnosed as having MCT were retrospectively examined. The mean age of the 80 patients who met the inclusion criteria was 30.60±10.5 years. Nine had infertility according to hospital records. Sixty-seven percent of these (n=6) had accompanying endometriosis and MCT was bilateral in 55.5% (n=5). Malignant degeneration was present in 6.25% (n=5), all were monodermal tumors. Malignant degeneration was more common among patients with larger diameter adnexal masses (9.1±2.9 cm) and in those of postmenopausal age. Tumor markers were within the normal range for patients who developed malignancy. Malignant degeneration was not present among infertile patients with endometriosis.

**Results:**

Although MCTs do not seem to negatively affect the ovarian reserve, infertility is prominent in patients with concurrent endometriosis. During assessment, concurrent endometriosis should be considered. Imaging findings, large adnexal masses, and postmenopausal period are important for the assessment of MCT concerning malignant degeneration. It should not be overlooked because tumor markers may be normal.

**Conclusion:**

MCTs can be present concurrent with endometriomas. In such cases, infertility is more distinct. In MCT malignant degeneration, mass diameter, complex mass internal structure, and postmenopausal status are important factors.

## Introduction

Mature cystic teratomas (MCTs) are the most common benign germ cell tumors during the adolescent and reproductive period. Histologically, they may include tissues differentiated from each of the three germ layers (ectoderm, mesoderm and endoderm). They are seen predominantly in the reproductive period; however, they are also seen in childhood and the postmenopausal period. MCTs account for 20-25% of all benign ovarian tumors and bilaterally rates are about 10-15% (1). The most common signs are abdominal pain and findings of a pelvic mass, but they can be detected incidentally as well.

Complications occur in 20% of patients with MCT (2). These complications include torsion, rupture, infection, and malignant transformation. Malignant transformation is quite rare and predominantly detected in the postmenopausal period, whereas other complications may cause undesirable reproductive outcomes concerning the age period when they occur. 

Our purpose in this article was to examine the cases of MCT that were diagnosed and treated in our clinic regarding the effects on fertility, rates of malignant transformation, and clinicopathologic features.

## Material and Methods

Files of patients who underwent surgery due to a prediagnosis of adnexal mass with a histopathologic diagnosis of MCT between April 2012 and August 2017 were retrospectively reviewed. Permission was obtained from the medical director of the hospital to access file information and computer records and from the ethics committee to use patient data. Patients who were diagnosed as having MCT after histopathologic evaluation and had sufficient demographic and medical information in their files were included in the study.

The inclusion criteria for our study were as follows: patients who were clinically evaluated, surgically treated, and diagnosed as having MCT in our hospital; patients who were not pregnant at the time of diagnosis; with a confirmed pathologic diagnosis of MCT; and adequate demographic and medical information in the case files. Diagnosis and treatment in another clinic, diagnosis during pregnancy, the presence of all other malignant and benign ovarian masses that received a different histopathologic diagnosis during the final evaluation, diagnosis such as tubo-ovarian abscess, and lack of sufficient information in the case file were set as criteria for exclusion.

Patient histories and file records were reviewed for patients with MCT, regarding the presence of any symptoms/reports of infertility before or during admission. In addition, the number of pregnancies and births were also taken into consideration. Imaging methods, tumor markers, and additional pathologies accompanying MCT were reviewed for all cases.

Concerning malignant transformation in MCT, the patient files were reviewed in terms of age, parity, family history, and malignancies of other organs.

## Result

The records of 93 patients who underwent surgery due to an adnexal mass and were diagnosed as having MCT between April 2012 and August 2017 were accessed. Thirteen of which were excluded mostly due to a lack of sufficient medical history and different histopathologic diagnoses. Eighty patients formed our study group and the mean age was 30.60±10.50 (range, 14-65) years. Seven patents (8.75%) were of postmenopausal age (age >45 years), 91.25% (n=73) were of premenopausal age (age ≤45 years). The most common symptom before admission was pelvic pain accompanied by abdominal distention in 76.25% (n=61) of the patients. The remaining 23.75% (n=19) were detected incidentally.

The most commonly performed imaging technique was abdominopelvic ultrasonography (USG). After an initial evaluation of all patients with USG, 13 patients were evaluated using computed tomography (CT), 33 patients with magnetic resonance imaging (MRI), and 2 patients with both CT and MRI.

The most frequently requested tumor markers to evaluate the malignancy potential of the adnexal mass were as follows: CA125 (n=80), CA19-9 (n=63). The mean value of CA125 was 24.23±16.10 IU/mL (normal reference range: 0-35 IU/mL); the mean value of CA 19-9 was 32.43±89.10 IU/mL (normal reference range: 0-35 IU/mL). When the cut-off value of 35 U/mL was accepted for both tumor markers, CA125 and CA19-9 were detected as high in 17.50% (n=14) and in 19.04% (n=12) of the patients, respectively. Both tumor markers were high in only 4.76% (n=3) of patients.

Fifty of the 80 patients were multiparous, the mean gravida was 1.78, parity was 1.12. Thirty patients were nulliparous. When all patients were evaluated according to the initial symptom, 9 had infertility. Among these 9 patients who underwent surgical treatment, apart from a dermoid cyst, endometriosis was found in six and one had hydrosalpinx (Table 1). The mean CA125 and CA19-9 levels of these 6 patients, who had infertility and diagnosed as having both MCT and endometriosis, was 39.16±16.60 IU/mL and 19.33±14.40 IU/mL, respectively. The CA125 level was above the threshold value in 4 of these six patients (66.67%); the CA19-9 level was also above the threshold in one patient. In 5 of these six cases, bilateral teratomas were present and the mean diameter of tumor mass was 5.10±1.80 cm according to the USG measurement.

Malignant degeneration was observed in 6.25% (n=5) of the patients. All malignant degeneration was monodermal-specialized tumor. Malignant degeneration was observed in 4.10% (3 of 73 patients) in the premenopausal period and in 28.57% (2 of 7 patients) in the postmenopausal period (Table 2). Among the patients with malignant degeneration, the mean mass diameter was 9.10±2.90 cm and no bilateral cases were observed. The mean values for tumor markers were 24.32±9.50 IU/mL for CA125, and 11.02±8.70 IU/mL for CA19-9, which were within normal reference values. Malignant degeneration was not observed in patients with infertility (Table 2).

## Discussion

MCTs are the most common benign ovarian neoplasms in the reproductive period that can originate from all three germ layers (ectoderm, endoderm, mesoderm), as well as from a single germ leaf (1). The second and third decades are the peak ages and mean ages are reported as 30 years (2). In our case series, the mean age of onset was 30.60±10.5 and 91.25% (n=71) of the patients were in the reproductive period. The most common symptoms at admission were abdominal pain and distention. Although abdominal pain is more prominent with adnexal mass torsion, both findings are common during the adolescent period when the pelvic volume is limited (3). USG is the first and most frequently used imaging modality with clinical examination in all age groups (4). The initial evaluation of all our patients was performed using pelvic USG. MRI and CT are the most frequently requested imaging modalities for further evaluation. The size and contents of the cyst, ratio of solid components, presence of mural nodule, and vascular blood flow assessment with Doppler USG are radiologic findings that provide important information concerning the treatment plan (4).

Concerning the evaluation of MCT, which is observed during the reproductive period and in a wide spectrum ranging from a simple ovarian cyst to a complex adnexal mass, tumor markers are laboratory tests that are frequently used. MCTs do not have a specific tumor marker. Tumor markers are also insufficient in demonstrating malignant degeneration (2,5). The most frequently requested tumor markers in MCTs are CA125 and CA19-9. Neither CA19-9 nor CA125 is diagnostic for MCT, although they are frequently used in the evaluation of adnexal masses of the reproductive period. MCTs present as adnexal masses, and elevated tumor markers may be helpful concerning the evaluation of the malignant potential of the mass with further examinations (5,6,7).

Although MCT cases are seen in adolescence and in the postmenopausal period, the incidence increases in the reproductive period. The current literature is more focused on endometriotic cysts and infertility; however, the effects of MCT on reproductive health are unclear. As far as we know, there are no studies showing that infertility is directly related with MCTs, as it is in endometriotic cysts. The incidence of endometriosis among infertile women ranges from 9 to 50%; the incidence of teratomas in infertile women is unknown (8,9). 

Unlike endometriomas, studies indicate that MCT does not reduce ovarian reserves (8,9,10,11). In our series, MCT was encountered in 9 patients during examinations for infertility, 6 of whom had accompanying endometriosis. In one patient, hydrosalpinx was also present. MCT was bilateral in 5 of these 9 patients. All patients with bilateral lesions (n=5) also had endometriosis. Endometriomas associated with MCTs are reported very rarely in the literature (12,13). Matalliotaki et al. (14) studied pathologies accompanying endometriosis in 1000 patients and reported that dermoid cysts were concurrently present in 1.2% of 295 cases of endometrioma.

Among the infertile patinets with MCT, the high incidence of accompanying endometriosis is the most characteristic feature of our case series. These results suggest that infertility is rather secondary to endometriosis. While evaluating patients with MCT who have infertility, bilaterality should be carefully investigated. Furthermore, foci of endometriosis should be thoroughly examined preoperatively with MRI or intraoperatively. 

Given the inflammatory, infiltrative progression of endometriosis, growth in MCT is slow, expansive, non-invasive, and non-inflammatory. Unlike MCT, endometriosis causes destruction and fibrosis of the ovarian cortex, leading to a decrease in the number of follicles (8,10). MCTs are mostly unilateral, but even when they are bilateral, fertility is not adversely affected by proper surgical intervention in the presence of healthy ovarian tissue (15). In MCT, postoperative recurrence (4.2%) is extremely low compared with endometriomas (45-50%) (16). Due to these developmental features of MCT, ovarian reserve and fertility is not as adversely affected. 

In the present study, no findings or problems related to fertility were found, according to the information collected from the patient records. In complicated large cysts (>8 cm), complications such as malignant degeneration, torsion, and rupture may develop; inappropriate surgical approach or spillage of cyst fluid may cause inflammation, irritation, and adhesions. In this case, if the preserved functional ovarian tissue is inadequate, subfertility and infertility may occur (8,10,17,18). If the cysts are small (<5 cm) and asymptomatic, they are unlikely to cause torsion and have a low potential for malignant transformation; we suggest that such special patient groups may be monitored until the completion of fertility. However, it is important to keep endometriosis in mind regarding patients with infertility.

Although dermoid cysts are slowly growing cysts, growth rates are increased by hormonal stimulation, especially by estrogen (19,20,21). Hormonal stimulation may be the reason for the high incidence of MCTs in reproductive age and also their tendency to become recognizable during pregnancy.

Complication (torsion, rupture, infection, malignant degeneration) rates were reported in the literature as up to 20% regarding MCTs, but no complications except malignant degeneration were observed in our series. Patients with adnexal masses and acute abdomen during pregnancy and emergency conditions were excluded in our study. Therefore, only patients who was selectively evaluated in a tertiary center were included in our case series.

Minimally invasive endoscopic approaches are important for the preservation of ovarian reserve and fertility in MCTs observed in the reproductive period and that are planned for surgical treatment because of the risk of complications (large mass diameter, rapid growth, pregnancy, risky imaging findings for malignant degeneration). Compared with endometriomas, MCT’s expansive and noninfiltrative growth facilitates surgical dissection and causes less destruction in the ovary (8,10). For this reason, fertility is less adversely affected by surgery performed for MCTs.

Monitoring may be an alternative approach in MCTs with small mass diameters, that are stable in periodic controls, and not complicated by internal structures (19). The most important factors that affect the surgical decision in the reproductive period are mass size, bilaterality, the risk of emergency surgical intervention such as for torsion and rupture, the likelihood of malignant degeneration, a complicated internal structure of the image, increased tumor markers, and fertility status (20,21,22).

The incidence of malignant degeneration in MCT is reported as 0.2-2%. The most common malignant degeneration, squamous cell carcinoma (SCC), accounts for 80% of all malignant degeneration in MCT (22,23). In our series, the rate of malignant degeneration was 6.25% (n=5) and all malignancies were monodermal specialized tumors. The causes of malignant transformation of monodermal specialized tumors are unknown. These tumors may cause different clinical symptoms according to their originating germ layer and hormonal activations (e.g., carcinoid syndrome, thyrotoxicosis).

Although in our patients with malignant degeneration, the size of the mass (mean 9.1 cm) and the frequency of postmenopausal age were consistent with the literature, the high rate of malignant degeneration, the histologic type of malignancy, and normal tumor markers were differences that distinguish our series from the literature.

MCTs are common benign ovarian tumors in the reproductive period. Unless they grow rapidly or cause torsion, rupture and malignant degeneration, they do not seem to affect ovarian reserve harmfully. Teratomas develop expansively and in a non-inflammatory nature, while endometriotic cysts grow in an inflammatory nature and infiltrative way; this distinction is responsible for this outcome. In addition, the better response given to surgical treatment, having conservative approach as a valid option, and low rates of recurrence are among the important features of MCTs. 

The most striking finding of our series is that regarding the patients in whom MCTs were concurrent with endometriomas, infertility was the initial symptom at admission.

An individualized conservative approach or delayed surgical treatment after completion of fertility may be appropriate and effective for patients with small and non-complex lesions in imaging studies, high expectation of fertility, and a high risk of losing significant ovarian reserve during surgery. However, if the mass is large, urgent surgical intervention should be considered because of the risk of torsion and rupture.

The outcome of ovarian protective treatment approaches is better than with endometriomas in terms of fertility when surgical excision is decided upon in MCT and appropriate surgical technique is selected. In the decision of exploratory surgical excision, patient age, clinical findings, possibility of emergency surgery, a complex internal structure in imaging modalities, elevated tumor markers, and reproductive expectations are determining factors.

## Figures and Tables

**Table 1:**
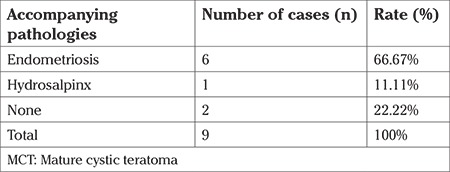
Accompanying pathologies in patients with MCT and infertility

**Table 2:**
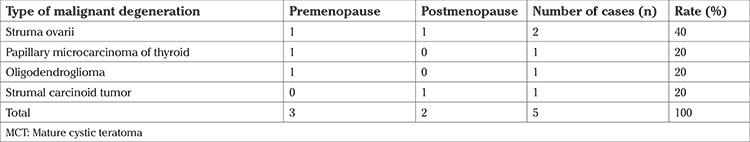
Histopathologic features and menopausal status of patients with MCT and malignant degeneration
